# Influenza Vaccine Effectiveness in the Elderly Based on Administrative Databases: Change in Immunization Habit as a Marker for Bias

**DOI:** 10.1371/journal.pone.0022618

**Published:** 2011-07-26

**Authors:** Travis S. Hottes, Danuta M. Skowronski, Brett Hiebert, Naveed Z. Janjua, Leslie L. Roos, Paul Van Caeseele, Barbara J. Law, Gaston De Serres

**Affiliations:** 1 Epidemiology Services, BC Centre for Disease Control, Vancouver, British Columbia, Canada; 2 School of Population and Public Health, University of British Columbia, Vancouver, British Columbia, Canada; 3 Department of Community Health Sciences, Faculty of Medicine, Manitoba Centre for Health Policy, University of Manitoba, Winnipeg, Manitoba, Canada; 4 Cadham Provincial Laboratory, Manitoba Health, Winnipeg, Manitoba, Canada; 5 Surveillance and Outbreak Response Division, Centre for Immunization and Respiratory Infectious Diseases, Public Health Agency of Canada, Ottawa, Ontario, Canada; 6 Institut national de santé publique du Québec, Québec, Québec, Canada; 7 Department of Social and Preventive Medicine, Université Laval, Québec, Canada; University of Texas Medical Branch, United States of America

## Abstract

**Background:**

Administrative databases provide efficient methods to estimate influenza vaccine effectiveness (IVE) against severe outcomes in the elderly but are prone to intractable bias. This study returns to one of the linked population databases by which IVE against hospitalization and death in the elderly was first assessed. We explore IVE across six more recent influenza seasons, including periods before, during, and after peak activity to identify potential markers for bias.

**Methods and Findings:**

Acute respiratory hospitalization and all-cause mortality were compared between immunized/non-immunized community-dwelling seniors ≥65years through administrative databases in Manitoba, Canada between 2000-01 and 2005-06. IVE was compared during pre-season/influenza/post-season periods through logistic regression with multivariable adjustment (age/sex/income/residence/prior influenza or pneumococcal immunization/medical visits/comorbidity), stratification based on prior influenza immunization history, and propensity scores. Analysis during pre-season periods assessed baseline differences between immunized and unimmunized groups. The study population included ∼140,000 seniors, of whom 50–60% were immunized annually. Adjustment for key covariates and use of propensity scores consistently increased IVE. Estimates were paradoxically higher pre-season and for all-cause mortality vs. acute respiratory hospitalization. Stratified analysis showed that those twice consecutively and currently immunized were always at significantly lower hospitalization/mortality risk with odds ratios (OR) of 0.60 [95%CI0.48–0.75] and 0.58 [0.53–0.64] pre-season and 0.77 [0.69–0.86] and 0.71 [0.66–0.77] during influenza circulation, relative to the consistently unimmunized. Conversely, those forgoing immunization when twice previously immunized were always at significantly higher hospitalization/mortality risk with OR of 1.41 [1.14–1.73] and 2.45 [2.21–2.72] pre-season and 1.21 [1.03–1.43] and 1.78 [1.61–1.96] during influenza circulation.

**Conclusions:**

The most pronounced IVE estimates were paradoxically observed pre-season, indicating bias tending to over-estimate vaccine protection. Change in immunization habit from that of the prior two years may be a marker for this bias in administrative data sets; however, no analytic technique explored could adjust for its influence. Improved methods to achieve valid interpretation of protection in the elderly are needed.

## Introduction

Elderly people (≥65 years) experience the greatest burden of severe complications from seasonal influenza, with more than 90% of influenza-related deaths estimated to occur annually in that age group [1], [2]. The US Advisory Committee on Immunization Practice first recommended routine influenza immunization for the elderly in 1964. The recommendation was predicated on the increased risk of severe influenza complications in the elderly, but evidence for vaccine benefit was acknowledged at that time to be based on extrapolation as follows:

“That influenza vaccine prevents mortality from influenza, particularly among the aged and chronically ill, is based upon inference. It is presumed that vaccine protection demonstrated in studies among younger persons is similar among the aged and chronically ill, the group at particular risk of death should they acquire the disease. It is further assumed that such protection against clinical disease serves to protect them also against mortality associated with epidemic influenza. No studies, however, have yet been reported which measure the efficacy of the vaccine in prevention of influenza-associated mortality.” [3]

Consequently, the recommendation to immunize elderly people against influenza became “grandfathered” into practice in North America, precluding placebo-controlled trials for ethical reasons. Only three randomized controlled trials (RCTs) have since assessed the efficacy of split trivalent inactivated influenza vaccine (TIV) in preventing laboratory-confirmed influenza in the elderly [4]–[6]. None was conducted in North America, none assessed vaccine protection against serious outcomes, most involved young/low-risk elderly, and all used serologic outcomes, which, as recently suggested, may overestimate influenza vaccine effectiveness (IVE) [7]. Two trials assessed serologically-confirmed influenza illness in community-dwelling elderly showing significant vaccine protection of 58% (95% confidence interval [CI] 26–77%;Netherlands,1991–92; [4]) and 65% (16–85%;Bangkok, 1998–99; [6]). One assessed protection among nursing home residents with a non-significant vaccine efficacy of 50% (−26–80%;Russia,1996–97; [5]).

The remaining evidence for the benefit of TIV in the elderly comes entirely from observational studies [8]–[31]. Fedson et al were among the first to report influenza vaccine effectiveness (IVE) against hospitalization and death in the elderly using the linked immunization and health outcome administrative databases for the population of Manitoba, Canada [9]. Fedson et al reported IVE against hospital admission for pneumonia of 37–39% and against all-cause mortality of 27–30% during the 1982–83 and 1985–86 seasons. Subsequent observational studies showed similarly impressive estimates of vaccine protection. In a 2002 meta-analysis, Vu et al [20] summarized IVE against pneumonia and influenza hospitalizations across nine studies as 33% (95%CI 19–47%) and against all-cause mortality across four studies as 50% (95%CI 45–56%). In most studies, adjustment for underlying health conditions further increased estimates of IVE [10], [19], [21], [23], [25], [28]—an effect believed to be the result of accounting for confounding by indication whereby higher-risk patients may be more likely to receive vaccine [32]. Estimates were interpreted as reliable because the same high level of vaccine protection was not measured outside periods of influenza circulation, notably in the summer [13], [16], [21], [22], [24], [28], [29].

More recently a Cochrane meta-analysis summarized similar estimates of IVE but questioned the quality and interpretation of available data [31]. Others have expressed similar skepticism in particular because substantial increase in TIV coverage among the elderly from <15% to >65% over the past four decades has not been accompanied by the kind of decrease in influenza-related mortality implied by such vaccine protection [33]. IVE against all-cause mortality of 50% is counter-intuitive given that the fraction of winter excess mortality attributed to influenza has never exceeded 10% over those four decades [33], [34]. Rather than under-estimating IVE through confounding by indication, observational designs may have instead substantially over-estimated IVE through *healthy user* (or *healthy vaccinee*) bias whereby elderly patients with poorer prognosis may be less likely to receive vaccine compared to the healthy [34], [35].

Jackson et al illustrated this effect in estimates for IVE against hospitalization and death that were paradoxically higher pre-season than during the period of peak influenza activity [36]. In follow-up case-control and cohort analyses, including detailed chart review, Jackson and others were able to adjust for specific markers of functional status and frailty and reported marked attenuation of vaccine benefit [37], [38]. Most influenza deaths were accrued in a small but special subgroup of under-immunized and incapacitated elderly experiencing acute decline.

The current study returns to the large longitudinal and linked Manitoba databases among the first used to assess IVE against serious outcomes in the elderly. We explore IVE across six more recent influenza seasons, including periods before, during, and after peak activity to identify potential markers for bias within administrative data.

## Methods

### 1. Ethics statement

The University of Manitoba Research Ethics Board and Health Information Privacy Committee (Manitoba Health) approved the study. Because anonymized administrative data were used in this study, individual consent was not required, per the Manitoba Personal Health Information Act and the evaluation of the Research Ethics Board and Health Information Privacy Committee.

### 2. Study population and setting

A cohort of community-dwelling adults ≥65years as of December 31 was assembled by linking data from the Manitoba Immunization Monitoring System (MIMS) and the Manitoba Centre for Health Policy data repository for September 1 to August 31 of each year from 2000–01 to 2005–06. Residents of personal care facilities were excluded. Adult immunization with date of administration has been routinely recorded in MIMS since 2000. For this study, individuals were considered immunized if their record indicated vaccine receipt by the date when 90% of the cohort had been immunized for a given study year (first or second week of November in all years). The remaining 10% of immunized individuals were excluded from the analysis in order to maintain a consistent immunized cohort throughout the entire influenza season. Physician visits, hospital claims, and vital statistics data were linked to vaccine records at the individual level.

### 3. Time periods

**Figure 1:**
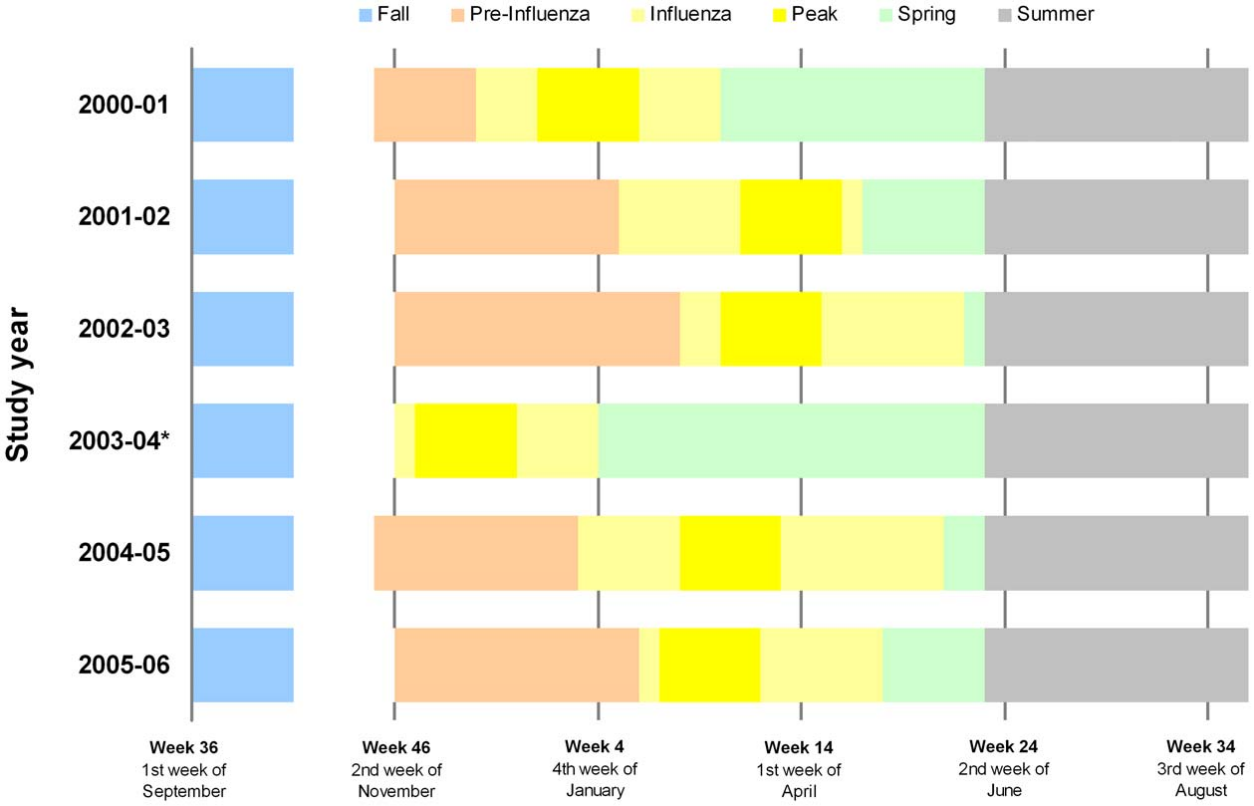
Timeline of seasonal periods of analysis, by study year. **Note.** Definition of periods as follows: Fall = five week period starting with first week of September; Pre-Influenza = interval from date by which 90% of elderly immunizations had been administered until onset of influenza period; Influenza = the interval between and including the first and last occurrences of at least two consecutive weeks with two or more influenza isolates reported; Peak = five week period including week with peak proportion of respiratory specimens positive for influenza +/− two weeks; Spring = interval from/including week after the influenza period to May 31; Summer = interval from/including first week of June to last week of August. * Pre-influenza period not defined in 2003–04 due to early season.

Weekly counts of influenza isolates recorded by Cadham Provincial Laboratory (Manitoba) defined discrete periods within study years including fall, pre-influenza, influenza, peak influenza, spring, and summer periods as shown in **Figure 1**. Trends in health outcomes were explored across these periods each year and in aggregate. For hospitalization, the five-week fall period beginning the first week of September each year (weeks 36–40) prior to the start of the annual immunization campaign was compared to the influenza period. IVE was calculated for this fall period by assigning study subjects their immunization status according to their actual immunization patterns during the coming season. This comparison was only possible for hospitalization since by definition those who died during the fall period could not be categorized based on subsequent immunization status. For all-cause mortality, comparison was thus based upon the pre-influenza period defined as the interval from the date by which 90% of elderly immunizations had been administered until the onset of the influenza period. Trends in relation to both the influenza and the peak periods were similar so only the former are presented.

### 4. Outcomes

Outcomes were all-cause mortality and hospitalization with pneumonia, influenza, or acute respiratory disease listed as the “most responsible” admission diagnosis. International Classification of Disease (ICD-9-CM and ICD-10) diagnostic codes defined these outcomes. The transition from ICD-9 to ICD-10 diagnostic coding took place April 1, 2004 in Manitoba and appropriate translations were made.

### 5. Covariates

The following covariates were considered: age (five-year groups with the eldest group consisting of those ≥85years), sex, socio-economic status (SES), urban residency, comorbid conditions, number of medical visits in the prior year (0–2, 3–9, ≥10 visits), number of influenza immunizations in the prior two years (none, one, or two), and pneumococcal immunization. Publicly available census data were used to sort approximately 20% of the Manitoba population into five ordered income classes, and study subjects were assigned to an income quintile, as a proxy for SES [39]. Urban residency was defined as residence in Winnipeg or Brandon. The previously validated Elixhauser Comorbidity Index [40] provided a measure of comorbidity based on the individual's hospital claims two years prior to the start date of each study year. Diagnostic codes from these hospital claims specified 30 different comorbidities [40]. The two-year sum Elixhauser Index was categorized as 0, 1–2, 3–5, 6–9, and ≥10. The number of prior medical visits was extracted from the physician claims database from September 1 of the previous year to August 31 of each study year, excluding the influenza immunization visit. In 2000 Manitoba introduced pneumococcal immunization for adults age ≥65years. For any given study year, pneumococcal immunization was defined if the vaccine was administered between January 1, 2000 and the first day of the influenza period.

### 6. Statistical analysis

Overall hospitalization and mortality rates were derived for each period and year as the number of events divided by the average living population over the period adjusted for the length of the period to enable comparisons. Where hospitalization and mortality rates are presented per year, they are also age-standardized using the 2006 population. Generalized estimating equations (GEE) computed aggregate rates across years accounting for year-to-year variability and adjusting for age. Aggregate predicted rates and odds ratios stratified by prior influenza immunization in the preceding two years were also derived by GEE adjusting for age, prior medical visits, and pneumococcal immunization.

Univariate and multivariable IVE estimates were calculated for each study period/year as one minus the odds ratio for a given outcome in the immunized compared to the non-immunized [41]. For both outcomes, new risk sets were assembled at the start of each week of IVE analysis, removing individuals who died in the previous week.

For yearly analyses, a logistic regression model was fit with adjustment for potential confounders and exploration of interaction terms. For propensity score analysis [42], [43], logistic regression models were fit with various combinations of covariates in order to calculate the probability of receiving an influenza vaccine. The model which yielded the highest *c*-statistic and lowest Akaike information criterion (AIC) value and which included all relevant covariates was selected; subjects were then matched 1∶1 based on current immunization status. Longitudinal GEE models were used to generate aggregate IVE estimates for each of the above approaches. We explored various correlation structures, and results were similar for auto-regressive 1 and exchangeable methods; exchangeable correlation structure was therefore used for all analyses. Because of an early start to the influenza season during the 2003–04 year, a pre-influenza period was not available for comparison of IVE against all-cause mortality that year. For that reason, the 2003–04 year was excluded from aggregate IVE analyses for all periods. For hospitalization, the 2003–04 year was excluded only from the pre-influenza period analysis since the fall period was the main referent for that outcome.

## Results

### Population characteristics

Assembled cohorts ranged in size from 139,185 in 2000–01 to 140,735 in 2005–06: 52%, 52%, 53%, 64%, 62%, and 64% received influenza vaccine each successive year (**Table 1**). These proportions are within 5–10% of vaccine coverage estimates from the Canadian Community Health Survey for 2000–01 (62%), 2003–04 (60%), and 2005–06 (71%) [44].

**Table 1:** Characteristics of study population by immunization status and study year, 2000–01 to 2005–06.

Year (N)	2000–01 (N = 139,185)			2001–02 (N = 139,665)			2002–03 (N = 139,534)			2003–04 (N = 139,186)			2004–05 (N = 140,069)			2005–06 (N = 140,735)		
	% in subgroup			% in subgroup			% in subgroup			% in subgroup			% in subgroup			% in subgroup		
Covariates	NI	I	VC %	NI	I	VC %	NI	I	VC %	NI	I	VC %	NI	I	VC %	NI	I	VC %
N	66228	72957	52	67257	72408	52	65754	73780	53	49618	89568	64	53232	86837	62	50490	90245	64
**Age group % (n)**																		
65–69	33 (21751)	23 (16537)	43	32 (21802)	22 (16157)	43	33 (21434)	22 (16032)	43	31 (15510)	24 (21530)	58	32 (17149)	24 (20453)	54	32 (16209)	24 (22097)	58
70–74	25 (16657)	26 (18642)	53	25 (16858)	25 (18361)	52	25 (16586)	25 (18526)	53	24 (12023)	25 (22177)	65	24 (12896)	24 (21234)	62	24 (12049)	24 (21603)	64
75–79	20 (13004)	24 (17725)	58	19 (13039)	24 (17534)	57	19 (12330)	24 (17630)	59	19 (9434)	23 (20365)	68	19 (10012)	22 (19401)	66	19 (9426)	22 (19760)	68
80–84	13 (8293)	16 (11783)	59	13 (8620)	17 (12034)	58	13 (8699)	17 (12771)	59	14 (7077)	17 (14996)	68	14 (7281)	18 (15260)	68	14 (7018)	17 (15516)	69
≥85	10 (6523)	11 (8270)	56	10 (6938)	11 (8322)	55	10 (6705)	12 (8821)	57	11 (5574)	12 (10500)	65	11 (5894)	12 (10489)	64	11 (5788)	12 (11269)	66
Median y (IQR)	73 (68–79)	75 (70–80)	n/a	73 (68–79)	75 (70–80)	n/a	73 (68–79)	75 (70–80)	n/a	73 (68–80)	75 (70–80)	n/a	73 (68–79)	75 (70–81)	n/a	73 (68–79)	75 (70–81)	n/a
**Sex**																		
Male	44	43	52	44	43	51	44	43	52	44	43	64	44	43	61	44	43	63
Female	56	57	53	56	57	52	56	57	53	56	57	65	56	57	63	56	57	65
**SES quintile** a																		
1	22	22	52	23	22	51	22	21	52	22	20	62	22	20	60	22	20	62
2	23	24	53	22	22	52	23	23	53	24	23	64	23	22	61	23	22	63
3	22	23	53	22	23	53	22	23	54	22	23	65	22	23	63	22	22	65
4	18	17	51	17	18	53	18	18	53	17	18	65	17	18	63	17	18	65
5	14	14	53	15	15	52	15	15	53	14	16	66	15	16	64	15	17	66
**Residency**																		
Urban	58	64	55	60	63	53	59	63	54	57	63	67	58	63	64	57	63	66
Other	42	36	48	40	37	50	41	37	51	43	37	61	42	37	59	43	37	61
**Number influenza immunizations in prior 2 seasons**																		
0	84	31	29	77	11	13	76	8	10	80	16	26	71	6	12	69	7	16
1	9	23	74	14	37	74	13	21	64	10	19	77	17	24	70	15	15	64
2	7	46	88	9	52	86	11	71	88	10	65	92	12	70	90	16	77	90
**Received pneumococcal vaccine since 1998**																		
Yes	1	10	89	10	55	86	12	58	86	17	73	90	27	79	84	31	85	84
No	99	90	53	90	45	37	88	42	37	83	27	39	73	21	34	69	15	30
**2-year sum of Elixhauser Index**																		
0	86	81	51	85	80	50	84	79	51	84	80	63	84	81	61	85	82	63
1–2	10	14	59	11	14	58	11	14	59	11	14	69	10	13	68	10	13	68
3–5	3	5	61	4	5	59	4	5	60	4	5	66	4	5	65	4	4	67
6–9	1	1	61	1	1	55	1	1	58	1	1	67	1	1	65	1	1	65
≥10	<1	<1	59	<1	<1	54	<1	<1	54	<1	<1	59	<1	<1	61	<1	<1	53
**Prior medical visits (1 y)**																		
0–2	31	11	28	29	10	27	29	10	28	31	11	39	32	12	39	32	13	42
3–9	44	48	54	45	49	54	45	48	55	44	49	67	46	52	65	46	53	67
≥10	25	41	65	25	41	63	26	41	64	25	40	74	22	36	73	22	34	74

**Note.** NI = non-immunized; I = immunized; VC = vaccine coverage; IQR = inter-quartile range; y = years; SES = socioeconomic status.

a Information to derive income quintile missing for 1% of the cohort in each study year).

Vaccine coverage tended to be higher with advancing age, in those residing in an urban area, and in people who had received prior influenza or pneumococcal immunizations or who had ≥10 medical visits in the prior year (**Table 1**).

The proportion that had received no influenza immunizations in the prior two years decreased each successive year from 56% in 2000–01 to 29% in 2005–06, while the proportion that had received two prior influenza immunizations increased each successive year from 27% to 55%. Each study year, those who had been immunized twice in the preceding two years had the highest current influenza immunization rates, always exceeding 85%; conversely, those who had received no dose in the preceding two years consistently had the lowest influenza immunization rates, never exceeding 30%.

The proportion of patients who had received pneumococcal vaccine steadily increased over the course of the study period, from 1% in 2000–01 to 31% in 2005–06 among the influenza non-immunized, and from 10% in 2000–01 to 85% in 2005–06 among the immunized.

### Hospitalization and mortality

**Figure 2:**
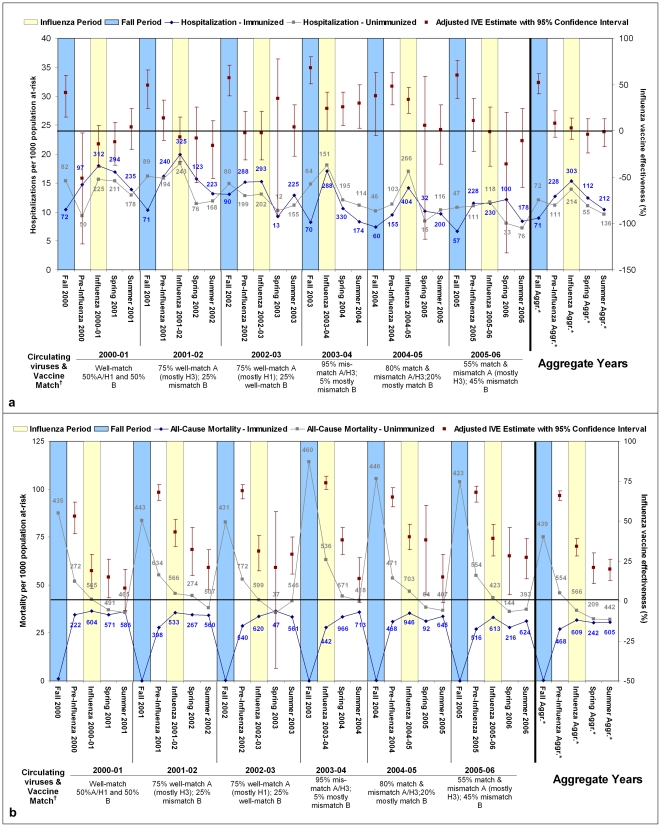
Hospitalization (pneumonia, influenza, or acute respiratory disease; panel a) and all-cause mortality (panel b) counts and rates by immunization status and influenza vaccine effectiveness estimates against hospitalization (panel a) and all-cause mortality (panel b), adjusted for age, prior medical visits, prior influenza vaccination, and prior pneumococcal vaccination, 2000–01 to 2005–06. **Note.** Numbers adjacent to rates represent numerator counts of hospitalizations per period and immunization status stratum; numbers in ‘Aggregate Years’ section represent median counts per period. There is no pre-period for 2003–04 owing to early start of influenza period. Hospitalization defined as admission with pneumonia, influenza, or acute respiratory disease listed as most responsible diagnosis. IVE = influenza vaccine effectiveness. * Aggr. = data aggregated across all 6 seasons 2000–01 to 2005–06 (2003–04 excluded for all-cause mortality). ^†^ Approximate influenza A versus B circulation (based on national summaries [45]) and match to vaccine components.

Counts and age-standardized rates for hospitalizations and all-cause mortality are plotted by period and year in **Figures 2a and 2b**, respectively. Hospitalization rates show a rise during influenza periods, lower in the periods before and after, for both immunized and non-immunized cohorts. However, hospitalization rates were consistently higher each year among the non-immunized compared to the immunized during the fall period, with greater variability in relative rates during other periods.

Mortality rates were highest in the non-immunized during the fall period each year and consistently higher in the non-immunized compared to the immunized across all periods, most notably in the period before influenza circulation.

### Influenza vaccine effectiveness

Crude IVE estimates and the effects of adjustment for influential covariates, matching by propensity scores, and stratification by immunization history are shown for each analysis period in aggregate in **Table 2** and for the influenza period of each year (with illustration of adjustment by individual covariates) in **Table 3**. Adjusted and propensity score-matched estimates were consistently higher than crude estimates.

**Table 2:** Aggregate influenza vaccine effectiveness against hospitalization and all-cause mortality estimated by GEE with adjustment, propensity score matching, and stratification: 2000–01 to 2005–06.

	HOSPITALIZATION e; IVE (95% CI)				ALL-CAUSE MORTALITY g; IVE (95 % CI)		
Covariate Adjustment	Fall	Pre-influenza f	Influenza	Summer	Pre-influenza	Influenza	Summer
**Crude**	26% (12, 38)	−13% (−30, 2)	−9% (−21, 2)	−7% (−22, 7)	42% (35, 48)	13% (6, 20)	4% (−6, 13)
**All covariates** a	54% (42, 64)	11% (−5, 24)	5% (−7, 16)	1% (−14, 14)	62% (58, 64)	31% (27, 36)	19% (13, 25)
**Select covariates** b	52% (40, 62)	8% (−7, 22)	3% (−9, 14)	−1% (−17, 13)	66% (63, 69)	34% (28, 39)	20% (13, 26)
**Matched propensity scores** c	46% (32, 57)	9% (−8, 24)	6% (−7, 18)	7% (−10, 22)	58% (53, 62)	30% (23, 36)	17% (8, 25)
**Stratified by number of influenza immunizations in prior 2 years:**							
0	−60% (−116, −19)	−63% (−115, −24)	−50% (−80, −26)	−50% (−88, −20)	18% (2, 32)	−13% (−30, 2)	−13% (−32, 3)
1	52% (32, 66)	10% (−16, 30)	5% (−15, 21)	18% (−5. 36)	59% (52, 65)	38% (30, 46)	21% (8, 32)
2	74% (67, 80)	37% (21, 49)	36% (23, 46)	22% (−1, 39)	75% (72, 79)	50% (44, 56)	39% (30, 48)
**Stratified by number of influenza immunizations in prior 2 years adjusted for all covariates** a							
0	−57% (−114, −15)	−48% (−88, −16)	−36% (−60, −16)	−39% (−67, −16)	33% (21, 43)	9% (−4, 20)	1% (−12, 13)
1	68% (54, 77)	35% (16, 50)	38% (25, 49)	41% (24, 53)	56% (49, 62)	34% (25, 42)	11% (−3, 23)
2	81% (75, 85)	41% (27, 53)	53% (43, 61)	37% (19, 51)	73% (70, 77)	46% (40, 52)	32% (22, 40)
**Stratified by number of influenza immunizations in prior 2 years and adjusted for select covariates** d							
0	−33% (−81, 2)	−34% (−73, −4)	−27% (−51, −7)	−31% (−60, −7)	21% (7, 33)	−3% (−16, 9)	−7% (−21, 6)
1	57% (40, 69)	17% (−6, 35)	9% (−9, 24)	24% (3, 40)	62% (56, 67)	42% (35, 48)	25% (14, 34)
2	74% (67, 79)	36% (21, 47)	36% (24, 46)	21% (0, 38)	76% (73, 78)	51% (45, 56)	40% (33, 47)

**Note.** IVE = influenza vaccine effectiveness; CI = confidence interval.

a All covariates include: age, sex, socio-economic status, urban residency, prior influenza immunization (two year), prior pneumococcal immunization, medical visits prior year, and Elixhauser index;

b Select covariates include: age, prior influenza immunization (two year), pneumococcal immunization, medical visits prior year;

c Matched on propensity scores derived based on all covariates: age, sex, socio-economic status, urban residency, prior influenza immunization (two year), prior pneumococcal immunization, medical visits prior year, and Elixhauser index;

d Select adjustment includes: age, prior pneumococcal immunization, medical visits prior year;

e Pneumonia, influenza, or acute respiratory disease listed as most responsible admission diagnosis;

f Pre-influenza period estimates for hospitalization do not include 2003–04;

g 2003–04 year excluded from all estimates related to all-cause mortality since the pre-influenza period constitutes the main comparison period for that outcome and was missing for 2003–04 owing to early influenza period.

**Table 3:** Influenza vaccine effectiveness against hospitalization and all-cause mortality during influenza periods, 2000–01 to 2005–06 with and without adjustment for multiple and select covariates.

Variable	2000–01 IVE (95% CI)		2001–02 IVE (95% CI)		2002–03 IVE (95% CI)		2003–04 IVE (95% CI)		2004–05 IVE (95% CI)		2005–06 IVE (95% CI)		Aggregate^e^ IVE (95% CI)	
	Hosp	Death	Hosp	Death	Hosp	Death	Hosp	Death	Hosp	Death	Hosp	Death	Hosp	Death
Crude	−24% (−48, −5)	5% (−6, 15)	−22% (−44, −3)	15% (5, 24)	−27% (−52, −6)	10% (0, 19)	−3% (−26, 15)	53% (47, 59)	9% (−6, 22)	15% (7, 23)	−6% (−33, 15)	17% (6, 27)	−9% (−21, 2)	17% (11, 22)
Age	−12% (−33, 6)	14% (4, 23)	−12% (−32, 5)	23% (13, 31)	−15% (−38, 4)	20% (10, 28)	4% (−17, 21)	56% (50, 61)	17% (3, 29)	23% (15, 30)	2% (−22, 22)	24% (14, 33)	0% (−9, 9)	23% (19, 28)
Prior influenza vaccine	−44% (−75, −18)	6% (−7, 18)	−36% (−69, −10)	37% (27, 46)	−11% (−43, 14)	28% (15, 38)	27% (5, 43)	75% (71, 79)	32% (16, 45)	40% (32, 47)	−3% (−39, 24)	38% (27, 47)	−7% (−22, 6)	30% (24, 35)
Prior pneumococcal vaccine	−22% (−45, −3)	7% (−5, 17)	−7% (−28, 11)	27% (17, 35)	−18% (−44, 3)	16% (5, 26)	−3% (−31, 19)	63% (57, 68)	17% (0, 31)	19% (9, 27)	−5% (−38, 19)	23% (11, 34)	−6% (−18, 5)	23% (17, 28)
Prior medical visits	2% (−16, 18)	19% (9, 28)	1% (−17, 17)	26% (17, 34)	−5% (−26, 13)	22% (12, 30)	15% (−4, 30)	61% (56, 66)	26% (13, 36)	28% (21, 35)	7% (−16, 26)	27% (18, 36)	10% (1, 18)	26% (22, 31)
Elixhauser index	−14% (−35, 4)	12% (2, 22)	−15% (−35, 3)	20% (10, 29)	−18% (−41, 2)	17% (7, 26)	−3% (−26, 16)	54% (48, 60)	13% (−2, 25)	19% (11, 27)	−2% (−27, 18)	21% (10, 30)	5% (−6, 14)	20% (15, 25)
All covariates^a^	−6% (−29, 12)	19% (8, 29)	−5% (−30, 15)	39% (30, 48)	−2% (−30, 20)	31% (20, 41)	15% (−12, 35)	72% (67, 76)	30% (13, 43)	38% (29, 45)	−7% (−46, 22)	35% (24, 45)	5% (−7, 16)	34% (29, 38)
Select covariates b	−14% (−39, 6)	19% (8, 29)	−7% (−33, 15)	43% (34, 51)	−2% (−31, 21)	31% (19, 41)	24% (0, 42)	74% (70, 78)	34% (19, 47)	40% (32, 48)	−1% (−38, 31)	39% (28, 48)	3% (−9, 14)	35% (29, 39)
Propensity score c	−16% (−46, 8)	10% (−5, 23)	4% (−28, 29)	45% (33, 54)	3% (−29, 27)	22% (6, 35)	15% (−21, 41)	67% (58, 73)	30% (9, 46)	38% (27, 48)	3% (−36, 31)	38% (24, 50)	6% (−7, 18)	30% (23, 36)
Stratified by number of influenza immunizations in prior 2 years d														
0	−32% (−66, −4)	−4% (−23, 12)	−6% (−57, 28)	4% (−29, 29)	−97% (−204, −27)	−16% (−56, 14)	41% (2, 65)	41% (16, 58)	−13% (−71, 26)	20% (−10, 42)	−57% (−177, 11)	−4% (−53, 29)	−36% (−60, −16)	−4% (−17, 7)
1	17% (−27, 46)	44% (27, 57)	−10% (−57, 23)	52% (40, 62)	−1% (−50, 32)	30% (9, 46)	24% (−25, 53)	73% (63, 80)	38% (12, 56)	36% (21, 48)	−21% (−105, 28)	44% (26, 57)	38% (25, 49)	42% (35, 48)
2	26% (−21, 55)	52% (37, 64)	14% (−36, 45)	57% (45, 66)	40% (16, 57)	49% (37, 59)	25% (−17, 52)	82% (78, 85)	49% (32, 62)	50% (40, 58)	41% (12, 60)	47% (34, 57)	53% (43, 61)	53% (49, 57)

**Note.** IVE = influenza vaccine effectiveness; CI = confidence interval; Hosp = hospitalization with pneumonia, influenza, or acute respiratory disease listed as most responsible admission diagnosis; Death = all-cause mortality; SES = socioeconomic status.

a Adjusted for all following covariates: age, sex, socio-economic status, urban residency, prior influenza immunization (two year), prior pneumococcal immunization, medical visits prior year, and Elixhauser index.

b Adjusted for select influential covariates of age, prior influenza (two year) and pneumococcal immunization and medical visits prior year.

c Matched on propensity scores, calculated based on age, sex, socio-economic status, urban residency, prior influenza immunization (two year), prior pneumococcal immunization, medical visits prior year, and Elixhauser index.

d Select adjustment includes pneumococcal immunization, medical visits prior year, and age.

e Data aggregated across all six seasons for hospitalization and across all seasons except 2003–04 for mortality.

For most years, during the fall period prior to influenza circulation, the rate of hospitalization in individuals who would remain unimmunized was much higher than in those who would become immunized, representing intrinsic differential between the ultimately immunized and non-immunized cohorts (**Figure 2a**). The calculated IVE was therefore high during that period and much higher than during the period of influenza transmission. Adjustment further exacerbated this bias, approximately doubling IVE estimates in the fall period prior to influenza circulation (**Table 2**). In stratified analyses, those who had received influenza vaccine in each of the preceding two years typically showed the highest IVE during the influenza period compared to those who had received none or one prior immunization (**Tables 2** **,** **3**).

IVE estimates against mortality were consistently higher than those against hospitalization. For all years, IVE against mortality was highest in the pre-influenza period (**Figure 2b**), a pattern that was also exacerbated with adjustment (**Table 2**). IVE estimates were lower during the influenza period but higher than those for the summer (**Table 2**). In stratified analyses, those who had received influenza vaccine in each of the preceding two years showed the highest IVE against all-cause mortality and those who had not previously received any doses derived the lowest IVE (**Tables 2** **,** **3**).

### Hospitalization and mortality by prior immunization history

**Figure 3:**
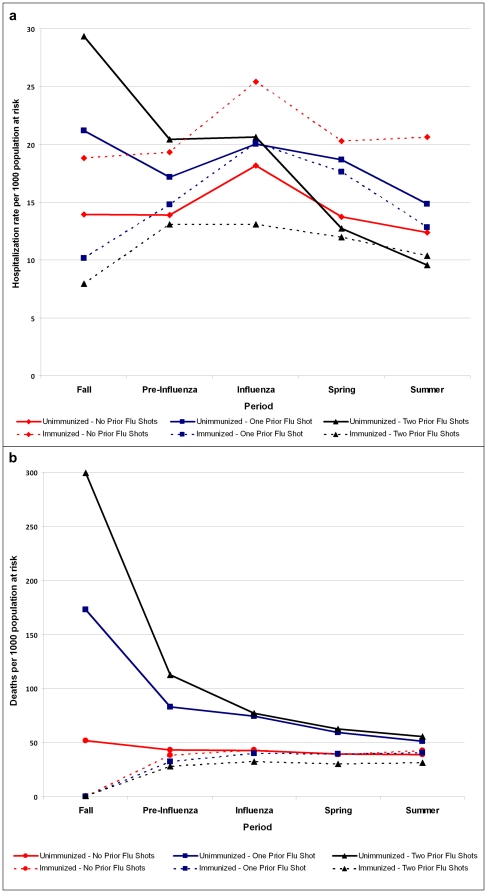
Aggregated hospitalization (pneumonia, influenza, or acute respiratory disease; panel a) and all-cause mortality (panel b) rates by prior & current immunization status, by period, 2000–01 to 2005–06. **Note.** Based on GEE model adjusting for age, prior medical visits, and prior pneumococcal vaccination, and accounting for year-to-year variation. 2003–04 excluded from pre-influenza period analyses for hospitalization and from all analysis periods for mortality owing to early start of influenza period. Hospitalization defined as admission with pneumonia, influenza, or acute respiratory disease listed as most responsible diagnosis.

Because stratification based on immunization in the previous two years showed an influence on IVE estimates, we plotted hospitalization and mortality rates and derived odds ratios stratified by historic and current immunization. These are shown as aggregate estimates for all study years adjusted for age, prior medical visits, and prior pneumococcal immunization in **Figures 3a/3b** and **Table 4**.

**Table 4:** Adjusted odds ratios for hospitalization and death by prior and current influenza immunization status.

	Adjusted a odds ratio (95% CI)			
Two year prior immunization history; current immunization	Fall	Pre-Influenza	Influenza	Summer
Hospitalizations b				
None; unimmunized	Ref	Ref	Ref	Ref
None; immunized	1.34 (1.00, 1.78)	1.38 (1.10, 1.74)	1.29 (1.12, 1.49)	1.35 (1.14, 1.60)
One; unimmunized	1.66 (1.25, 2.20)	1.29 (1.04, 1.61)	1.24 (1.06, 1.47)	1.29 (1.06, 1.57)
One; immunized	0.73 (0.56, 0.94)	1.1 (0.90, 1.29)	1.10 (0.97, 1.25)	0.99 (0.86, 1.15)
Two; unimmunized	2.34 (1.82, 2.99)	1.41 (1.14, 1.73)	1.21 (1.03, 1.43)	1.01 (0.80, 1.27)
Two; immunized	0.60 (0.48, 0.75)	0.89 (0.76, 1.04)	0.77 (0.69, 0.86)	0.78 (0.68, 0.90)
Deaths (All-cause)				
None; unimmunized	n/a	Ref	Ref	Ref
None; immunized	n/a	0.76 (0.65,0.89)	0.94 (0.84, 1.05)	1.07 (0.67, 1.18)
One; unimmunized	n/a	1.73 (1.57,1.92)	1.66 (1.51, 1.82)	1.30 (1.17, 1.45)
One; immunized	n/a	0.68 (0.60,0.76)	0.89 (0.81, 0.96)	1 (0.92, 1.09)
Two; unimmunized	n/a	2.45 (2.21,2.72)	1.78 (1.61, 1.96)	1.35 (1.21, 1.51)
Two; immunized	n/a	0.58 (0.53,0.64)	0.71 (0.66, 0.77)	0.79 (0.73, 0.85)

**Note.** CI = confidence interval. 2003–04 could not contribute to pre-influenza period for hospitalizations and was excluded from all periods for mortality owing to early start to influenza season that year.

a Adjusted for age, prior pneumococcal immunization, and medical visits prior year.

b Pneumonia, influenza, or acute respiratory disease listed as most responsible admission diagnosis.

The elderly who were previously immunized and continued to receive vaccine experienced the lowest hospitalization and mortality rates across all analysis periods. The previously unimmunized who received vaccine had the highest hospitalization rates during the influenza period. Those forgoing immunization who had twice previously received vaccine had the second highest hospitalization rates during the influenza period, but their rates were already greater compared to other groups during the fall and pre-influenza period, with steady decline thereafter. Mortality rates in those forgoing immunization despite prior receipt showed the same pattern of decline from a fall excess compared to the immunized in whom mortality was lower and more stable across all analysis periods, regardless of prior immunization history.

Odds ratios to quantify the risk between immunized and unimmunized elderly according to prior immunization history are shown in **Table 4** **,** with the consistently non-immunized as referent. For those previously immunized, forgoing immunization was associated with a significantly higher likelihood of both hospitalization and death that was evident in advance of the influenza period. Conversely, those who were previously and again immunized were at significantly lower risk at all times. Those choosing to be newly immunized were at significantly higher risk for hospitalization (but not death) before and during the influenza season. Given the disproportionate contribution of previously versus never immunized to the total cohort, results suggest an overall tendency to over-estimate IVE against severe outcomes in the elderly.

## Discussion

This study returned to the administrative databases that were among the first to show substantial reduction in serious influenza outcomes among immunized elderly. In revisiting the Manitoba database, we exposed similar evidence for bias that others have found, with the most pronounced but implausible effects (i.e., differences between immunized and non-immunized groups) observed in the elderly prior to influenza circulation or even vaccine distribution. We showed that change in immunization habit relative to the preceding two years may be a readily accessible and recognizable marker for this bias.

We defined immunization status based on vaccine receipt by the first two weeks of November for a given year (the period by which 90% of those immunized had received vaccine). Based on that consistent categorization, we found that the ultimately non-immunized group had higher hospitalization and mortality rates than the immunized before the period of influenza circulation. Given the impossibility of a true vaccine effect during the fall period prior to vaccine distribution, multiple analytical methods were evaluated based on the degree by which they could reduce this obvious positive bias. As in other studies, adjustment by standard regression and propensity score matching only exacerbated bias, substantially increasing IVE estimates against hospitalization and death before and during the influenza period.

Simonsen et al have suggested three criteria to identify residual bias in observational studies of IVE [33]. The first criterion specifies that a null vaccine effect should be observed during pre-influenza periods. As already discussed, this was not shown in our study. Secondly, IVE should be least pronounced during seasons when vaccine components are poorly-matched to circulating strains. As shown in **Figures 2a/2b**, we found no obvious correlation between vaccine match and IVE estimates. Lastly, IVE estimates should be most pronounced for more specific outcomes (e.g., pneumonia and influenza hospitalization) and less so for non-specific outcomes (e.g., all-cause mortality). In our study, as in others, the reverse was true.

In combination, this framework signals substantial bias in our estimates of IVE derived using classical methods and a well-established administrative database similar to that typically used by others reporting substantial vaccine protection. Further stratification based on prior influenza immunization revealed at least one obvious indicator of this bias: rates of hospitalization and death varied with change in immunization habit relative to the preceding two years. We showed high rates of hospitalization and death among those forgoing immunization after having twice consecutively received vaccine—an excess that was greatest even before the influenza period began—highlighting a nuanced form of healthy user bias. These individuals may represent the “healthy user recently turned unhealthy”, whose acute failure to receive vaccine is a marker for imminent decline, thereby contributing to hospitalization and death counts in the unimmunized and spuriously inflating estimates of vaccine protection. Baxter et al have also recently shown that forgoing immunization predicts death in those who had received vaccine in the previous five years but predicts survival in patients who had never before received vaccine [46]. We were able to show these same effects based on change from just two years of influenza immunization habit, suggesting that alteration in vaccine behavior is a robust predictor of death, albeit one which could not, through multivariable analysis, correct the bias described in this paper. In the opposite direction, seeking vaccine after forgoing for several years may reflect a change in health/risk status resulting in disproportionate contribution to hospitalization within the immunized group—a classic form of confounding by indication spuriously lowering IVE estimates. These forms of bias influence IVE estimates in opposite directions and may differentially affect hospitalization and death as serious outcome indicators, adding to the complexity of analysis based on administrative data sets.

Because of the numerous limitations outlined in this paper, we resisted citing specific IVE estimates in the discussion of our findings. Overall, compared to estimates reported by Fedson et al two decades ago in the same population and with similar methods, our aggregate estimates for vaccine protection during the influenza period are slightly higher against all-cause mortality but lower against hospitalization [9]. We have presented analyses using a specific hospitalization outcome (pneumonia, influenza, or acute respiratory disease listed only as most responsible admission diagnosis) which may not have captured all hospitalizations attributable to influenza; in preliminary analyses, however, less specific hospitalization outcomes were also explored (e.g., all-cause hospitalization), and trends in IVE across periods and methods were similar to those using the more specific definition. In this study, we applied the unconventional approach of assigning immunization status during the fall periods—*before* the campaign started—based on a future exposure, with the intent of illustrating baseline differences between those eventually immunized and those never immunized in a given season. This approach would have classified those who died between the fall analysis period and the availability of vaccine as *un*immunized. Although these individuals did not have the opportunity to receive vaccine in the subsequent season, any effect of such misclassification on rates and IVE estimates would be small: of the 408 persons who were classified as unimmunized and hospitalized during the fall across the six study seasons, only 12 (3%) died before the start of the pre-influenza period.

While administrative data sets are recognized as efficient methods to estimate IVE, the validity of estimates derived in that way appears highly questionable. Our study illustrates the profound non-comparability of immunized and non-immunized individuals which is not corrected, but rather is exacerbated, by adjustment for standard confounders. Rather than continuing to assert potentially misleading evidence for protection, we see a few possible directions for research enhancement. More detailed exploration of covariates associated with acute decline is clearly needed. One example, which we were unable to explore in this study, may be a measure of suddenly stopping long-term medication for a chronic condition. Instrumental variables as a proxy for frailty could also be used [47], however, a reliable proxy not associated with the outcome has been challenging to identify in studies of IVE [48]. Case-control or nested case-control studies such as those conducted by Jackson et al have been successful in addressing functional status and frailty but such studies are labor-intensive and less amenable to annual repeat [36], [37]. Other methods have also been proposed [46], [49]–[51], but as long as original RCT evidence for vaccine protection in the elderly remains scant, it will be impossible to compare findings from observational studies against a reliable gold standard or to interpret true vaccine benefit. It may therefore be time to reopen the discussion for a properly designed RCT, with appropriate antiviral treatment, data monitoring, oversight, and stopping rules. Given that placebo-controlled trials may be ethically controversial in the elderly, randomization to standard versus enhanced (e.g., adjuvanted) formulations may be more acceptable.

Until then, our findings add to the growing uncertainty about whether current influenza vaccines provide needed protection to the elderly. Insomuch as the elderly suffer the most severe consequences of influenza infection, resolving whether current vaccine options offer benefit and otherwise advocating for improved approaches should be priorities for the influenza immunization program.
